# The patient’s voice: an exploratory study of the impact of a group self-management support program

**DOI:** 10.1186/1471-2296-13-65

**Published:** 2012-06-29

**Authors:** Sharon Johnston, Hannah Irving, Karina Mill, Margo S Rowan, Clare Liddy

**Affiliations:** 1 C.T. Lamont Primary Health Care Research Centre, Bruyère Research Institute, Ottawa, Ontario, Canada; 2 Department of Family Medicine, University of Ottawa, Ottawa, Ontario, Canada; 3 Rowan Research and Evaluation, Ottawa, Canada

**Keywords:** Chronic disease, Self-management, Diabetes, Community health

## Abstract

**Background:**

Given the potential value of self-management support programs for people with chronic diseases, it is vital to understand how they influence participants’ health attitudes and behaviours. The Stanford Chronic Disease Self-Management Program (CDSMP), the most well-known and widely studied such program, is funded in many provinces and jurisdictions throughout Canada. However, there is little published evidence on its impact in the Canadian health-care system. We studied participants’ reactions and perceived impacts of attending the Stanford program in one Ontario health region so we could assess its value to the health region. The study asked: What are participants’ reactions and perceived impacts of attending the Stanford CDSMP?

**Methods:**

This mixed methods exploratory study held four focus groups approximately one year after participants attended a Stanford program workshop. At the beginning of each session, participants filled out a survey on the type and frequency of community and health resources used for their self-management. During the sessions, a moderator guided the discussion, asking about such things as long-term impact of the program on their lives and barriers to self-management of their chronic conditions.

**Results:**

Participants perceived diverse effects of the workshop: from having a profound impact on one area to affecting all aspects of their lives. A change in physical activity patterns was the most prominent behaviour change, noted by over half the participants. Other recurrent effects included an improved sense of social connection and better coping skills. Barriers to self-management were experienced by almost all participants with several dominant themes emerging including problems with the health system and patient-physician interaction. Participants reported a wide variety of resources used in their self-management, and in some cases, an increase in use was noted for some resources.

**Conclusions:**

Self-management support is, at its core, a complex and patient-centred concept, so a diversity of outcomes to match the diversity of participants should be expected. As these interventions move into different target populations and communities, it is essential that we continue to explore through multiple research methods, the effects, and their meaning to participants, ensuring the optimal investment of resources for the very individuals these interventions aim to serve.

## Background

The alarming increase in chronic diseases and their burden on individuals and families has led many developed countries to consider redirecting part of their care from medically focused diagnosis and treatment to patient-focused prevention and self-management [1]. As one of the most innovative reforms, self-management support (SMS) arose from the disproportionate amount of time people spend managing chronic conditions outside of the care system compared to interacting with health-care providers [2,3].

SMS focuses on the patient, encourages collaborative goal setting, and builds self-efficacy [4] to enable patients to better manage their health in partnership with health-care providers [5-7]. SMS interventions are proliferating across populations with myriad approaches, including weekly group workshops [8], individual community-based peer support [9], and multidisciplinary primary-care-based health coaching [10]. Such interventions can increase patient’s confidence in managing their conditions, and have been shown to reduce inappropriate use of health-care systems and improve clinical outcomes for conditions such as diabetes in some populations [11,12]. Nevertheless, many questions remain about the degree, duration and effect of these programs [13]. Given the potential value of SMS programs, it is vital to better understand how they influence participants’ health attitudes and behaviours.

The Stanford Chronic Disease Self-Management Program (CDSMP) [8] is the most well-known and widely studied SMS program, evaluated in countries around the world [14,15]. Developed by the Stanford Patient Education Centre to support people living with chronic conditions, the standardized curriculum and program implementation – as well as the wealth of accessible, validated tools – have made it the most accessible program for clinical interventions and research applications. The U.S. Centres for Disease Control and Prevention conducted a recent meta-analysis of randomized control trials and longitudinal studies of participants in the CDSMP which showed sustained mild to moderate effects on a number of outcomes from the standardized evaluation toolkit. These effects include improved and sustained confidence or self-efficacy to manage one’s disease and symptoms, and decreased social and role limitations [12]. In addition to the quantitative findings presented in the meta-analysis, a recent qualitative study of the intervention has found that participants in the Stanford program value the group experience and sense of social connection that the group environment fosters [13].

Although the CDSMP is funded in many provinces and jurisdictions throughout Canada, there is little published evidence on its impact within our health-care system. In response to this gap in knowledge, we helped implement the Stanford CDSMP in the Champlain health region, located in northeastern Ontario. The program offered free six-week group workshops in both rural and urban centres, recruiting participants with chronic conditions and their caregivers through referral from health care providers and advertising in local media and community venues and meetings.

Given the lack of evidence of effect in Canadian communities and recognizing the importance of the community context in the development and maintenance of self-management practices [16], an ongoing evaluation was built into the implementation of the program. This paper describes the results of a study that asked: What are participants’ reactions and perceived impacts of attending the Stanford CDSMP?

## Methods

This mixed methods exploratory study used focus groups to elicit participants’ reactions and perceived impacts of attending the Stanford CDSMP. We also piloted a survey on health system and community resource use to assess the program’s effect on the health region. The study received approval from both The Ottawa Hospital and Bruyère Continuing Care institutional Research Ethics Boards.

### Sampling and recruitment

All 36 people who participated in the first four pilot CDSMP workshops were contacted by phone by a member of the research team approximately one year after the workshops, inviting them to participate in focus groups with other members of their workshop. These initial workshops took place in the fall of 2009 in Ontario’s Champlain Local Health Integration Network, home to 1.2 million Ontarians – covering a major urban centre, and smaller rural towns – and reporting a disease burden similar to the rest of the province.

Twenty people – 16 women and four men – agreed to create four focus groups. Reasons for non-participation were lack of time, illness and/or lack of interest due to incompletion of the workshop. Eighteen participants had a chronic condition about which they consulted a health-care provider; the other two were caregivers for family members with chronic conditions.

### Data collection

Two focus groups took place in their original rural settings and two of the groups were held in an urban setting. The groups met in the same location as the pilot workshops or in a place nearby that was accessible. The meetings were moderated by a member of the research team with the study coordinator observing and taking field notes. The moderator guided the discussion using the same template for each session. Questions included:

· What has been the greatest impact of the self-management workshops on your ability to self-manage your disease?

· What kinds of barriers have prevented you from achieving full success in self-managing your disease to the extent that you would like to manage it?

· What kinds of things are helping you to achieve success in self-managing your disease?

Focus groups were stopped after four as theme saturation was reached. Signed informed consent was obtained from all participants prior to audio-recording the sessions.

### Surveys on resource use

At the beginning of each focus group session, the participants filled out a survey designed by the team to capture the type and frequency of community and health resources used for their self-management over the past six months. Participants were asked to report on their use of more than 30 resources for self-management or health information, including health-related specialists such as doctors, physiotherapists, nutritionists, and naturopaths as well as resources such as friends and family, the internet, libraries, grocery store clerks, etc. We asked how often each resource had been used, offering a scale ranging from never to once a week over the past 6 months. We also asked whether they increased, decreased, or did not change their use of each resource compared to before their participation in the CDSMP.

### Data analysis

#### Qualitative

The recordings from each focus group were transcribed verbatim and coded by a member of the research team using NVIVO™8 software. Summaries of themes noted across one or multiple transcripts were reviewed by the entire team, with at least two team members reviewing each. Following this review, the entire team met to confirm identification of theme content and meaning as well as to identify themes recurring within a single group, or across multiple groups, and to identify disconfirming data.

#### Quantitative

The self-management resource use survey results were entered into SPSS® software to generate descriptive statistics about the percentage of the sample with a prevalence of use for each resource as well as whether or not this use had increased, decreased, or remained the same following their participation in the Stanford program.

## Results

### Degree of impact

Participants reported diverse effects of the CDSMP workshop, ranging from having a profound impact on one particular area to affecting all aspects of their lives to having no effect. A few participants noted the effect was immediate, when they realized they were not alone. Thinking back to the workshops, one participant said:

"*It was the first night when my whole world changed. … The first thing we did was talk about feelings and, you know, I was amazed that we all had the same feelings of guilt, of depression, of frustration,* etc.*, it didn’t matter what the illness was. And that, to me, was really empowering, like just to know that I wasn’t isolated, I wasn’t the only one (FG2).*"

For others, however, it was a while before they were aware of any effect and for some, it was not until faced with another new health challenge that they realised the workshop had had an effect. As one participant stated, “I took the course and it did help me a lot. And actually it is kicking in a lot more now because I was recently diagnosed with type II diabetes. I have learned how to use what we got in the course to cope with it” (FG4).

### Change in physical activity

A change in physical activity patterns was the most prominent difference in behaviour, noted by more than half the participants. This did not necessarily imply an increase in the rate of physical activity. Rather, within this single behaviour, the effect was quite diverse. For some, it was finding new ways to exercise based on ideas shared during the workshop. For instance, one participant adapted her physical activities to maintain a satisfactory level of activity: “Rather than doing the exercise class I have been doing for several years [which] I can’t do because of the pain, I switched totally to chair exercise” (FG1). For others, a change in physical activity meant being creative in finding the time to exercise, including doubling up on activities, such as what one participant called “aerobic cleaning” (FG4) or taking part in physical activity programs at the same time and location as other family members to reduce the number of trips made and time spent traveling (FG1).

For most who noted exercise as a benefit, integrating walking into their lifestyles was the single most reported result. The effect was quite varied: simply starting, increasing the minutes significantly or changing how or with whom they walked. One participant said: “I just recently found another lady who loves to walk, so we do what we call ‘the square,’ it’s an eight-kilometre walk” (FG3)*.* Another participant explained how she integrated walking into her daily routine: “[My son] got me the cutest little dog and… and I have to walk him… I’m not afraid to go outside anymore. I used to protect myself from the outside because there were so many triggers out there. I was really very worried and now I go outside and I walk him for two hours a day…and I think in order to be able to even do that, I think the group was very helpful” (FG2).

### Change in personal interactions

Another recurrent effect noted by participants in all four focus groups was an improved sense of social connection. Many participants noted a decrease or elimination of a sense of isolation through an increased sense of common ground. Recalling a fellow male peer expressing his emotions in the group another male participant said, “I think men are kind of scared to express how they feel… it’s not manly. Some of the things that came out at those sessions certainly helped me. I kind of felt good about it, that I’m not the only ‘sissy’…That sort of opened up a lot of emotions. And it helped me a bit” (FG2). Another participant expressed similar feelings, “I got a lot of comfort in coming just knowing that there are other people having major issues like you…you never felt alone because you knew everyone else had a problem as well” (FG3).

Multiple participants reported a significant effect of the intervention being a change in social interaction patterns linked to new activities. One participant who had started walking noted: “I just said, ‘Okay, I’ll do what I can do and I’m going to go out there and see what happens,’ and I’ve met all kinds of people, you know, and we go out for dinner and it’s just wonderful” (FG4).

Many participants reported a significant effect on how they interact with friends and family and how they receive self-management support from those around them. One participant noted “[the workshops] certainly changed my experience with my son… I find now I am able to say to him ‘I’m not feeling well today… can you pick up the slack?’” (FG2)*.*

### Improved coping skills

A final recurrent effect across focus groups was improved coping skills. There were two distinct approaches to coping better with the burdens of living with a chronic condition. Several participants reported developing a more proactive attitude in how they lived and experienced their conditions to enable them to make change happen. A participant described this change in attitude stating, “[the greatest impact of the workshop was] knowing you can manage whatever is going on in your life…it’s not hopeless, you can set goals and reach them” (FG4). Others seemed to develop an acceptance of their situations, which made it easier to cope with their conditions: “When people started sharing, I thought, ‘you know it’s not just me, it’s not just me’. Everyone else is doing the best they can with what’s going on” (FG3). Likewise another individual expressed:

"*I’m starting to accept it more than before…it was like ‘This is it, it’s not reversible’. But somewhere in the back of your mind you keep saying ‘Well maybe there’s this’…you start praying…and then you sort of accept it, and then live with it, and do the best we can. That’s one of the things that I got out of this…I went to the course and three of four weeks later I started to look at it differently (FG2).*"

### Facilitators to self-management

Many participants reported discovering facilitators in managing their health after participating in the workshops. The most frequently reported type was support from friends and family or connecting with another person who had a chronic condition and a level of empathy or understanding that helped them in their self-management process. One participant said, “I liked the goal setting with me it was the walking. I had marked down I would do 60 min or whatever of that walking for that week and I did do that, you know, with my husband…and it’s fun” (FG4). A few participants mentioned the availability of allied health in the community as a helpful resource in improving their self-management. “I also had the occupational therapist and physiotherapists and they were both very useful,” one participant noted. “I also have a chiropractor. … But I had to get them myself” (FG1).

### Unmet expectations

For some participants, the benefits they had hoped to achieve at the onset did not materialize. Some said they wanted more direct and immediate assistance or relief. “I expected more input in the beginning, to tell me that it wasn’t just going to be how I am going to cope with it, [but that] maybe they could help me with the pain,” one participant said (FG1). Others found suggestions offered in the group to be unrealistic for their situation: “Some of the suggestions are a little hard to get…like your physiotherapist. It is hard to get an appointment and then there’s the cost. I just kind of wished there was ideas that were within our means. My doctor is excellent…and my pharmacist is excellent…But as far as other types of resources, they’re just hard to get at” (FG3). Another participant had wanted opportunities to practice self-management activities while at the workshops. She stated, “I was hoping like maybe one session would be half an hour of yoga with a specialist to really get us into that rather than just reading the book, having diagrams of the pictures of the exercises… I wanted someone to show me” (FG1).

### Barriers to self-management

Barriers to self-management were experienced by almost all participants and included a range of issues. However, several dominant themes emerged. Problems with the health system were the most frequently cited barriers and, of those, patient-physician interaction was reported the most. Some participants noted the physician’s lack of knowledge or links with SMS resources in the community to be a key barrier. As one participant said: “[Physicians] do not know too much about what [programming] is going on in the vicinity” (FG3). Others cited insufficient time to discuss their personal health agendas with their physicians or a lack of expertise related to their case. One participant noted: “We need more doctors who are willing to undertake geriatric cases … [and] who have experience with geriatric cases” (FG1). Wait times to see specialists and lack of access to allied health were also cited by several participants. One participant shared her frustration with accessing mental health programming in particular: “There’s no programs for us anymore. If I think I’m ready now to go to a group therapy, it’s over a year waiting period … [but] I need that now, not in 18 months’ time. I may be dead by then” (FG4).

Many participants reported other issues, such as financial barriers and accessing additional programs or resources in managing their health. Lack of transportation and such symptoms as pain or fatigue were also cited as limiting factors in the ability to undertake self-management behaviours. One participant shared the impact her symptoms were having on her activity levels: “I was always extremely active and I just don’t have the energy anymore. I’ve had many friends call me to say… ‘We’re going for a walk.’ I said, ‘Well today you will have to count me out, I just can’t.’ So that is disappointing that that happens” (FG3).

### Resources used

The participants reported a wide array of health system and community resources used to manage their health and 20 to 30% of participants noted an increase in use of some resources, particularly nutrition-related ones, following participation in the workshops. Participants reported living in their communities for an average of 28 years and the majority (70%) reported having access to the internet in their homes. Additional survey results reported in Table 1.

**Table 1:** Resources used in participant’s self-management

**Resource**	Use post workshop* (n = 20)			Program’s impact on use post workshop** (n = 20)			
	used	not used	n/a	increased	decreased	no change	n/a
*Health care resources*							
Family physician	85%	10%	5%	5%	15%	65%	15%
Dietician, nutritionist, etc.	35%	50%	15%	20%	-	45%	35%
Pharmacist	85%	15%	-	15%	5%	80%	-
*Social support*							
Family	85%	5%	10%	15%	5%	70%	10%
Friends	75%	15%	10%	10%	5%	70%	15%
*Physical activity resources*							
Group fitness	40%	35%	25%	15%	5%	25%	55%
Other physical activities	40%	25%	35%	25%	10%	10%	55%
*Educational resources*							
Internet	60%	30%	10%	20%	-	50%	30%
Bookstore	65%	10%	25%	25%	-	45%	30%
*Food and nutrition resources*							
Waiter/waitress	40%	35%	25%	10%	-	50%	40%
Health food store staff	60%	20%	20%	30%	-	40%	30%
Other food and nutrition resources	50%	15%	35%	30%	5%	20%	45%

* Participants were asked to report on usage after the workshop within the 6 months prior to completing the survey.

** Participants were asked whether participation in the CDSMP had led to an increase, decrease or no change in their use of that resource.

### Limitations

The small sample size may limit the generalisability of the survey findings. However, the sample included both rural and urban participants and more than half of the original participants in the first four CDSMP workshops. The survey was designed by the team to capture a range of resources across populations, however the tool focused only on frequency of use and may not have adequately captured the types of changes made by participants. Further, participants were asked to recall information on a variety of practices over a year’s time, which might diminish the reliability of the results.

## Discussion

The focus groups and surveys offered a valuable opportunity to explore CDSMP participants’ perspectives and experiences of self-management in the year after the intervention. The results highlight that this complex intervention had a wide range of outcomes perceived by participants. Even the most reported effect of changed physical activity involved significant variation in what it meant to individuals.

### Social connectedness

Studies of the CDSMP have shown that many participants experience a positive outcome in the social connections established through the group setting [17,18]. Recent examination has also shown that a positive perception of the group aspect is most associated with other positive outcomes [13]. A recurrent theme through our focus groups was also the effect of the social connection that occurred. However, there was an interesting distinction between the immediate sense of losing the feeling of isolation that the group intervention created and reports of increased social connection often linked to other behaviour changes resulting from the group, such as meeting people after starting a walking program. Thus the effect of social connection was more than just being a part of a group in the workshop and, for some, had a more sustainable effect of better connecting them with their communities.

### Community support

The frequent link between self-management behaviours and changes in social interactions, highlights the importance of social relationships, as well as the surrounding environment in the participants’ ability to adopt and sustain self-management. Rogers has suggested that the importance of changes in social relationships might be undervalued, with an undue focus on evaluating the individual patient’s activation or emphasis on medical management of conditions [19]. Our findings reinforce the need to continue to explore the mechanism of effect of SMS interventions related to changes in social interactions within the larger community, not just in the health system. At present, there is a paucity of studies reporting the effect of programs in changing communities’ support for self-management. However, this might be a potential outcome for programs and suggests a need for approaches that do not focus exclusively on the individual participant.

Tools such as the Community Illness Resource Scales [19] are seeking to measure changes in support from such relationships while the health education impact questionnaire (known as the heiQ) aims to measure social integration and support [20]. Very few participants reported clinical outcomes or health care utilization pattern changes as important effects of participating in this intervention, yet these are often the justification for implementing these programs and the targets for evaluation [21]. Ensuring that the patient’s perspective is included in the evaluation and design of evaluation of such patient-centred interventions may help identify differences between stakeholders in outcomes of interest.

### Disconnect with health system

The Theory of Planned Behavior recognizes the effect of attitudes, subjective norms and perceived ability on shaping behaviour [22]. In applying this theory to the study results, it is interesting to note that many of the perceived resources and opportunities to engage in new self-management behaviours arose from interactions with people in the community, such as new friends, and not through relationships within the health care system. In fact, the most frequently reported barriers related to lack of self-management support within the health system. This finding was mirrored during an environmental scan of the region’s self-management support resources completed the year before the CDSMP workshops. That scan found that providers recognize the need to break down silos of care and disconnects between the community resources where a person lives and the health system in which they seek support [16].

A disconnect between the principles of self-management learned in supportive interventions and the lack of support for self-management experienced with physicians has been shown previously [23,24]. The recent meta-analysis of results of the CDSMP [12] showed that short-term improvement in communication patterns with physicians captured on participant survey results were not sustained at the one-year mark. This suggests a continued need to follow up on the potential effect on the health system and providers as well as communities of patients seeking support and opportunities to engage in self-management. If these interventions target individuals rather than their health providers, will they lead to a slowly evolving health system responding to changing patients’ expectations? Our findings also support the need for multi-pronged strategies to improve patient self-management targeting individuals, communities and the health system.

### Community and health resource use

In our survey, participants reported a wide variety of resources used in their self-management. Further, all the resources listed experienced a net increase in the percent of participants reporting utilizing them. Family and friends, as well as traditional health care providers (physicians and pharmacists), were the most frequently accessed. The limited change in use of resources such as physicians and pharmacists after participation in the CDSMP, compared to greater or similar proportion of participants reporting increased use of community resources, such as nutrition resources and bookstores, might reflect that participants already had sufficient use of health system resources for their needs, or a lack of capacity of those resources to be used more frequently. Alternatively, the limited change in provider usage may have been due to a perceived lack of value in increased use as participants noted health system interactions as barriers to self-management.

The survey results show other physical activity resources utilization increasing as much as utilization of nutrition related resources and family physicians. However, our qualitative findings showed change in physical activity behaviour was most often reported as the most significant effect of the program. Similarly, increased utilization of family and friends was reported by only fifteen percent of participants. Nonetheless the qualitative results revealed the significance of the changed quality of interactions with friends and family for this group of participants. This reinforces the importance of exploring the impact of interventions on the nature of interactions with support resources, as well as the frequency of interactions or visits.

The pilot survey findings suggest a pattern of increased use of diverse community resources. This deserves further study to better understand how informal community resources might serve to support patients in their efforts to self-manage, particularly if health system resources have limited capacity to increase visits.

### Recommendations for future research

The qualitative methods of this study allowed us to hear the patient perspective on important effects of this program and highlighted a need for further evaluation efforts to adopt a comprehensive approach to measuring outcomes. The results of our exploration of the patient perspective suggested that the significance of some outcomes or changes for individuals might not be adequately captured in our traditional survey measures using the standard Stanford measurement tools. The most important outcomes for some participants would barely register on a survey as a change in scale of minutes exercised. Initiating two blocks of walking or switching exercise types might not be a significant enough increase in actual time of physical activity to capture a change, despite its importance to the individual. More nuanced measures of physical activity such as the International Physical Activity Questionnaire [25] might better capture changes in physical activity in a variety of domains of daily life such as in household chores. Additionally, a more in-depth survey of the effect of community support for self-management experienced as an outcome of such programs such as the Community and Illness Resource Scale [19] might better capture meaningful effects of the program. A growing number of validated tools are available to assist researchers in measuring a wider range of outcomes of chronic disease self-management support programs. Exploratory qualitative assessments with different research populations may guide selection of survey instruments most likely to capture outcomes specific to the study population and assist in efficient evaluations as many of these tools are significantly longer than the Stanford tool set [8].

The need to show an effect on medical or health care spending outcomes – such as decreased use of health system resources, which might be deemed important to funding stakeholders (unpublished observations) – may bias evaluations to measure and therefore report on outcomes most directly linked to these effects. This may undervalue the effect of the intervention from participants’ perspective.

However, program funders and participants may have different priorities for investing resources, whether financial or time, in such interventions. Thus there is a tension between understanding the effect of such a program on different stakeholders’ desired outcomes and understanding the effect of such a program. This tension becomes greater as more resources are invested in interventions and implementers are encouraged to show an effect of their programs.

Valuable resources are required to evaluate such programs as both surveys and focus groups require substantial commitments from participants and implementers. Compromises on what outcomes to focus on for evaluation purposes have to be made.

## Conclusions

Self-management support is, at its core, a complex and patient-centred concept. Our results highlight the importance of exploratory qualitative research to capture potentially diverse outcomes and engage patients as new approaches to care continue to emerge. As interventions like the Stanford CDSMP move into different target populations and communities, it is essential that we continue exploring the effect and meaning of these effects to the participants to ensure the optimal investment of resources for the very individuals these interventions aim to serve.

## Abbreviations

SMS, self-management support; CDSMP, Stanford Chronic Disease Self-Management program.

## Competing interests

The author(s) declare that they have no competing interests.

## Authors’ contributions

SJ and CL conceived the study and MR collected the focus group data. All authors worked on the analysis and interpretation of the data collected through focus groups and surveys as well as participated in drafting the article and final reviews.

## Pre-publication history

The pre-publication history for this paper can be accessed here:

http://www.biomedcentral.com/1471-2296/13/65/prepub
